# Does sexual experience affect the strength of male mate choice for high‐quality females in *Drosophila melanogaster*?

**DOI:** 10.1002/ece3.8334

**Published:** 2021-11-10

**Authors:** Chelsea S. Sinclair, Suriya F. Lisa, Alison Pischedda

**Affiliations:** ^1^ Department of Biology Barnard College Columbia University New York USA

**Keywords:** behavioral plasticity, courtship, *Drosophila melanogaster*, female body size, male mate choice, mating history

## Abstract

Although females are traditionally thought of as the choosy sex, there is increasing evidence in many species that males will preferentially court or mate with certain females over others when given a choice. In the fruit fly, *Drosophila melanogaster*, males discriminate between potential mating partners based on a number of female traits, including species, mating history, age, and condition. Interestingly, many of these male preferences are affected by the male's previous sexual experiences, such that males increase courtship toward types of females that they have previously mated with and decrease courtship toward types of females that have previously rejected them. *D*. *melanogaster* males also show courtship and mating preferences for larger females over smaller females, likely because larger females have higher fecundity. It is unknown, however, whether this preference shows behavioral plasticity based on the male's sexual history as we see for other male preferences. Here, we manipulate the sexual experience of *D*. *melanogaster* males and test whether this manipulation has any effect on the strength of male mate choice for large females. We find that sexually inexperienced males have a robust courtship preference for large females that is unaffected by previous experience mating with, or being rejected by, females of differing sizes. Given that female body size is one of the most common targets of male mate choice across insect species, our experiments with *D*. *melanogaster* may provide insight into how these preferences develop and evolve.

## Graphical abstract

In *Drosophila*, many male courtship preferences are modified by the male's sexual experience. Here, we tested whether male mate choice for larger, higher‐fecundity females was affected by past experience mating with, or being rejected by, females of different sizes. We found no effect of sexual experience, with all males showing a courtship preference for larger females.

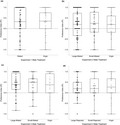

## 1 INTRODUCTION

Traditional theories of sexual selection predict that females should be the choosier sex in most species because of their higher investment in offspring production and lower variance in fitness (Andersson, 1994; Bateman, 1948). Although this is often the case, there is increasing evidence in various species that males will show courtship and mating preferences for certain females over others when given a choice (Amundsen, 2000; Bonduriansky, 2001; Edward & Chapman, 2011). This is not surprising, as males can experience significant costs associated with courtship and mating, and females often vary in quality, with some females producing more and/or higher quality offspring than others (Edward & Chapman, 2011). When females differ in quality and the costs associated with courtship are high, males may increase their fitness by directing courtship and mating efforts toward higher quality females (e.g., Arbuthnott et al., 2017; Edward & Chapman, 2012).

In the fruit fly, *Drosophila melanogaster*, male mate choice has been found in response to several female traits. *D*. *melanogaster* males are more likely to court or mate with healthy females compared with females infected with a parasite or pathogen (Khan & Prasad, 2013; Wittman & Fedorka, 2015), younger females compared with older females (Nandy et al., 2012), virgin females compared with mated females (Baxter et al., 2015; Cook & Cook, 1975; Siegel & Hall, 1979), and conspecific compared with heterospecific females (Dukas & Baxter, 2014; Shahandeh et al., 2020). Additionally, multiple studies have shown male mate choice based on female body size, with males preferentially courting or mating with larger females compared with smaller females (Baxter et al., 2015; Byrne & Rice, 2006; Long et al., 2009). This preference for large females is likely adaptive, as body size is positively correlated with fecundity in *D*. *melanogaster* (Lefranc & Bundgaard, 2000; Long et al., 2009), so males that mate with larger females may father more offspring.

Interestingly, many male courtship preferences are modified by the male's previous sexual experiences. For example, *D*. *melanogaster* males that had been rejected by a mated female subsequently reduced courtship toward mated females, as did males that had previously mated with a virgin female (Dukas & Dukas, 2012). In contrast, males that had mated with a simulated‐mated female (i.e., a virgin female treated with the pheromones of mated females) subsequently increased their courtship toward mated females (Dukas & Dukas, 2012). Males can similarly use pheromones to discriminate between *D*. *melanogaster* females of different ages and will suppress courtship toward the type of female that had previously rejected them (Balaban‐Feld & Valone, 2018; Ejima et al., 2005). Additionally, *D*. *melanogaster* males that had been rejected by a female from the closely related species *D*. *simulans* subsequently courted *D*. *simulans* females less than inexperienced males (Dukas, 2004; Dukas & Dukas, 2012), as did *D*. *melanogaster* males that had previously mated with a conspecific female (Dukas & Dukas, 2012). These courtship modifications can occur rapidly, with male courtship behavior changing significantly after only a single mating (Dukas & Dukas, 2012) or a single rejection episode lasting 10 min (Balaban‐Feld & Valone, 2018), 30 min (Dukas & Dukas, 2012), or 60 min (Dukas, 2004). Taken together, these results indicate that male fruit flies can readily refine their courtship preferences in response to a previous successful or unsuccessful courtship encounter.

While it is clear that past sexual experiences can affect male mate choice for some female traits, it is unclear whether this is the case for preferences based on female body size. Female body size is one of the most common targets of male mate choice in insects (Bonduriansky, 2001), so experiments with *D*. *melanogaster* provide the opportunity to investigate how these preferences develop. Although mating with a large female instead of a small female could potentially increase male fitness due to the larger female's higher fecundity, individual *D*. *melanogaster* males court large females with varying intensity and a substantial number court or mate with small females even when large females are present (e.g., Byrne & Rice, 2006; Long et al., 2009). The sexual history of experimental males is often not controlled, so this variation might reflect the experience‐based plasticity we see for other male preferences. Consistent with this hypothesis, *D*. *melanogaster* males that had recently been sperm/ejaculate‐depleted by mating multiple times had a stronger preference for large females compared with control males that did not have their mating history manipulated (Byrne & Rice, 2006). This increased male choosiness in sperm‐depleted males was attributed to the higher costs of mating associated with diminished resources (Byrne & Rice, 2006), but it is possible that the different mating histories of experimental and control males also contributed.

In this study, we altered the sexual experiences of *D*. *melanogaster* males and tested whether this affected the strength of male mate choice for larger females. We compared the courtship and mating preferences of sexually inexperienced males with males that had their sexual experience manipulated in one of four ways: (a) males that had mated with random‐size females 2 days before male mate choice trials, (b) males that had mated with small or large females 2 days before male mate choice trials, (c) males that had mated with a small or large female immediately before male mate choice trials, and (d) males that had been rejected by a small or large female immediately before male mate choice trials. A single mating is sufficient to significantly reduce seminal fluid protein amounts in males, but these levels are readily restored following a period of sexual inactivity (Sirot et al., 2009). As a result, the first two experiments gave males a 2‐day recovery period between the experience phase and the male mate choice trials to minimize any potential confounding effects of sperm and/or ejaculate depletion in mated males that could affect the strength of male mate choice (Byrne & Rice, 2006). The second set of experiments had the male mate choice trials immediately following the experience phase to control for the possibility that past experiences may not continue to affect male courtship behavior 2 days later (e.g., Siegel & Hall, 1979). Regardless of the experiment, sexually inexperienced virgin males consistently showed robust courtship preferences for large females, and the strength of male mate choice did not change with any of our sexual experience manipulations.

## 2 MATERIALS AND METHODS

### 2.1 *D. melanogaster* stock maintenance and culture methods

The *D*. *melanogaster* flies used in this study came from the LH_M_ population. LH_M_ is a large, wild‐type, outbred population of *D*. *melanogaster* that had been held under identical conditions in the lab for over 700 generations at the time of our experiments (Rice et al., 2005). The population is reared on a 2‐week, discrete generation lifecycle in 25 mm diameter vials containing 5–10 ml of cornmeal/molasses/yeast medium. Each generation on day 12 post‐egg ~1800 breeding individuals are lightly anesthetized using CO_2_ and are then distributed among 56 vials seeded with 6.4 mg of additional live yeast. 2.5 days later, the adult flies are transferred into fresh, unyeasted vials for 18 h, and the eggs that are produced are manually culled to a standard density of 150–200 eggs per vial. All flies were maintained at 25°C and 50–70% humidity on a 12‐h light:12‐h dark cycle.

### 2.2 Creating large and small female fruit flies

We created small and large females for male mate choice trials by manipulating larval density using the methods developed by Byrne and Rice (2006). We placed 16 pairs of sexually mature male and female fruit flies from the LH_M_ population into embryo collection cages (3.75 cm diameter by 5.8 cm high; Genesee Scientific) fitted with 35 mm petri dishes containing food medium for females to oviposit on for approximately 18 h. To create large females, exactly 50 eggs were collected from a petri dish and placed into a vial containing 10 ml of food. To create small females, we collected 100 eggs from a petri dish and placed them into a vial containing 1 ml of food. This protocol for altering female body size creates large females that are approximately twice the size of small females (Byrne & Rice, 2006) and thus are visually distinguishable without magnification. Flies in the small treatment developed slower than flies in the large treatment, so the small female vials were set up 1 day before the large female vials to ensure that the large and small female flies were the same age post‐eclosion for all experiments. 9–10 days after the egg setup (for the large and small females, respectively), the females were collected as virgins within 6 h of eclosion using light CO_2_ anesthesia. Large and small females were held separately in vials containing food medium at a density of 10 females per vial until the male mate choice trials. Before the experiments began, the vials were checked for larvae to ensure female virginity.

### 2.3 Rearing experimental males

Males for each experimental trial were randomly collected from vials reared under standard LH_M_ conditions at a density of 150–200 eggs per vial in vials containing 5–10 ml of food medium. Nine days after the egg setup, males were collected as virgins within 6 h of eclosion under light CO_2_ anesthesia and placed in groups of five in vials containing a small amount of food medium. Males were collected at the same time as the large and small virgin females to ensure that all flies were same post‐eclosion age for each experiment.

### 2.4 Altering male sexual experience

To determine whether the strength of male mate choice for large females was affected by the male's past sexual experience, we altered the sexual history of our experimental males in four separate experiments. This sexual experience phase was conducted either 2 days before the male mate choice trials, when flies were 3 days old post‐eclosion (Experiments 1 and 2), or immediately before the male mate choice trials (Experiments 3 and 4). After the sexual experience phase, we conducted all male mate choice trials when the experimental flies were 5 days old post‐eclosion. In reference to the normal LH_M_ lifecycle, the male sexual experience phase at 3 days post‐eclosion (12 days post‐egg) corresponds to the time at which these males would be transferred from their developmental vial into fresh vials with females. Similarly, the male mate choice trials at 5 days post‐eclosion (14 days post‐egg) occur within the last 24 h of the lifecycle, at a time when mating rates are elevated (Long et al., 2010). As a result, the sexual experience phase and the male mate choice trials both occur at biologically relevant times within the LH_M_ lifecycle.

#### 2.4.1 Experiment 1: Mating with random‐sized females 2 days before male mate choice trials

Theory predicts that males should use their past encounters with females to evaluate their own attractiveness and modify their degree of choosiness accordingly, such that successful mating experiences lead to males with stronger courtship and mating preferences (Fawcett & Bleay, 2009). As a result, we first tested whether male mating status (virgin or mated) affects the strength of male mate choice for large females. For this experiment, we mated males to random‐sized females 2 days before our male mate choice trials to ensure the males were not sperm/ejaculate depleted during our trials. We collected random‐sized females as virgins from the same vials as our experimental males, under standard LH_M_ culture conditions (150–200 eggs in vials containing 5–10 ml of food). To create “mated” males, five virgin males were placed with 10 random‐sized virgin females into vials containing food medium when the incubator lights came on and these flies were left for 1 h at 25°C. We used a mass‐mating protocol for the experience phase instead of directly observing matings because this less labor‐intensive approach allowed us to increase the sample size of males available for the experimental trials. Additionally, our preliminary work indicated that all of the males successfully mated at least one time in 28/30 trials under these conditions using a 30‐min interaction period, so here we increased the interaction time to 1 h to ensure that all (or nearly all) males mated. While it is possible that some males mated with more than one female during this time, the 2‐day recovery period allows time for any multiply‐mated males to restore their sperm and/or ejaculate supplies (Sirot et al., 2009). We also concurrently set up a virgin male treatment as a control by placing five virgin males into a vial containing food medium, but no females, for 1 h at 25°C. At the end of the hour, the mated male vials were lightly anesthetized using CO_2_, the females were discarded, and the males were returned to the vials in groups of five. Flies from the virgin male treatment were also lightly anesthetized with CO_2_ at this time. All males were then held at 25°C for 2 days until the male mate choice trials (see below). In total, we set up 46–47 mate choice trials per treatment for Experiment 1.

#### 2.4.2 Experiment 2: Mating with small or large females 2 days before male mate choice trials

We next tested whether the phenotype of the female that the male had previously mated with (i.e., large or small) affects the strength of male mate choice for large females 2 days later. With the exception of the size of the females used for the mating experience, Experiment 2 was conducted identically to Experiment 1. To mate our experimental males, five virgin males were placed with either 10 large or 10 small virgin females in vials containing food medium when the incubator lights came on and were left for 1 h at 25°C to create “large‐mated” and “small‐mated” male treatments. We do not expect any difference between the two treatments in the number of males that mated during this experience phase, as past work in the LH_M_ population has shown that large and small virgin females are equally receptive to mating (Byrne & Rice, 2006), which is consistent with data collected in the present study (see Experiment 3 below). Although some males might have mated with more than one female during this experience phase, the mated females would have been consistent in phenotype (i.e., both large females or both small females), and the 2‐day recovery period provides time for any multiply mated males to restore their sperm and/or ejaculate supplies (Sirot et al., 2009). We also set up a virgin male treatment concurrently, as in Experiment 1. The females were discarded after this experience phase and the males were held in groups of five at 25°C for 2 days until the male mate choice trials. In total, we set up 54–57 male mate choice trials per treatment for Experiment 2.

#### 2.4.3 Experiment 3: Mating with small or large females immediately before male mate choice trials

To control for the possibility that past mating experience may not continue to affect male behavior after 2 days (e.g., Siegel & Hall, 1979), our third experiment mated males to either small or large females immediately before the male mate choice trials. Because males would not have time to replenish their sperm and/or ejaculatory reserves before the male mate choice trials, we directly observed flies during the experience phase to ensure that the males only mated a single time. As soon as the incubator lights came on, virgin males were aspirated individually into a vial containing food medium and either a single large or a single small virgin female that had been aspirated into the vial the previous day. Foam plugs were pushed down into the vials to create approximately 1–2 cm of interaction space. We observed these vials for 30 min at room temperature to ensure that the males mated; any males that did not mate in this time were discarded. There was no significant difference between the number of males that mated in the large experience treatment (60/64) and the small experience treatment (57/65; Chi‐square test: *χ* ^2^ = 1.403, *p* = .24), or in the time it took males to mate with the large or small females (large females: mean = 9.82 min, 95% confidence interval = 8.40–11.23 min; small females: mean = 11.23 min, 95% confidence interval = 9.77–12.68 min; *t*‐test: *t* = 1.377, *df* = 115, *p* = .17). Males were allowed to finish mating uninterrupted, and the females were then removed from the vials using an aspirator and discarded. We also set up a virgin male treatment at the same time by aspirating males individually into vials containing food medium, pushing foam plugs down into the vials to create 1–2 cm of space, and leaving the males at room temperature while the large‐mated and small‐mated sexual experience occurred. All male mate choice trials using these virgin, small‐mated, and large‐mated males began within 30 min of the completion of the mating experience phase. In total, we set up 36–37 male mate choice trials per treatment for Experiment 3.

#### 2.4.4 Experiment 4: Rejection by small or large females immediately before male mate choice trials

To test for the effect of an unsuccessful sexual experience, our last experiment measured the strength of male mate choice in males that had been rejected by either a small or large female immediately before. We used nonvirgin, and thus sexually unreceptive, large and small female flies as our “rejecting females” for this sexual experience phase. To obtain these nonvirgin small and large females, we set up additional vials with manipulated egg densities at the same time that we set up the vials for our experimental small and large females. However, instead of collecting the rejecting females as virgins, we allowed them to eclose and mate within their developmental vial. We transferred all of the eclosed flies into new vials containing fresh food medium 2 days before our male mate choice trials to ensure the adults had adequate food resources. Past work in LH_M_ indicates that 96–99% of the large and small rejecting females will have mated in the developmental vial at this time (Long et al., 2010). The morning of our experiment (and about 1 h before the incubator lights came on), we collected small and large rejecting females individually into vials containing a small amount of food medium under light CO_2_ anesthesia. These females were given 1 h to recover at room temperature before the rejection sexual experience phase began.

At this time, a single virgin male was added to each vial using a mouth aspirator, such that each male was placed with either an individual large rejecting female or an individual small rejecting female. Foam plugs were pushed down into the vials to create 1–2 cm of interaction space and we observed these vials for 30 min at room temperature to ensure that the males courted, but did not mate, the rejecting females. None of the males mated with the rejecting female during this experience phase (*N* = 50 per treatment) and we only saved males that courted the female at least 10% of the time (≥3 min). The number of males that met this threshold did not differ significantly between the large (38/50) and small (40/50) treatments (Chi‐square test: *χ* ^2^ = 0.233, *p* = .63). At the end of the rejection phase, the rejecting females were removed from the vials using an aspirator and discarded. We also set up an inexperienced virgin male treatment at the same time using the protocol for the virgin treatment in Experiment 3. All male mate choice trials using these inexperienced, small‐rejected, and large‐rejected males were started no later than 30 min after the completion of the rejection experience phase. In total, we set up 30–31 male mate choice trials per treatment for Experiment 4.

### 2.5 Male mate choice trials

All male mate choice trials were conducted when the experimental males and females were 5 days old post‐eclosion. The evening before each mating observation, one large virgin female and one small virgin female were aspirated into a vial containing a small amount of food medium and returned to the 25°C incubator overnight. Once the incubator lights had turned on the next morning, we aspirated a single male (from one of the treatments described above) into each vial containing one large and one small female and pushed a foam plug down into the vial to create an interaction space of approximately 1–2 cm. For each experiment, males from the different treatments were distributed evenly among observers who were blind to the treatments, with each individual observing 10 vials at a time. The vials were observed for 30 min, and minute‐by‐minute courtship data were collected. Specifically, courtship was recorded for each minute in which the male performed a courtship song (in which the male extends and vibrates one or both wings while in proximity to a female) or an attempted copulation (in which the male curls his abdomen toward a female but is unsuccessful at copulating), and we noted which female these courtship efforts were directed toward. When a male courted both the large and the small female within the same minute, both courtship instances were recorded for that minute. We focused on singing and attempted copulation because these forms of courtship were easier to track during our observations compared with other courtship components like chasing, licking, and tapping (Spieth, 1974). If the male mated a female during the observation period, we recorded any courtship that occurred up to and including the minute the mating began; if the male did not mate, we recorded any courtship that occurred throughout the 30‐min observation period. All observations were completed within 2 h of the incubator lights turning on.

All four of our experiments used the male mate choice trial methods described above, with a few exceptions. Because Experiments 1 and 2 did not have a sexual experience phase immediately before the male mate choice trials, we were able to conduct two or three replicates back‐to‐back within 2 h of the incubator lights turning on. The different male treatments were evenly split between replicates and the flies for later replicates were held at 25°C until their male mate choice trials began. We found no significant effect of replicate on male courtship preferences (see below) in either experiment (Wilcoxon/Kruskal–Wallis tests; all *p* > .11). Additionally, due to laboratory restrictions imposed by the COVID‐19 pandemic, a subset of the data for Experiments 3 and 4 were obtained from video recordings of male mate choice trials. These recordings were made using an iPhone 11 (Apple) held horizontally over the vials by an Arkon Pro Phone Stand (model number HD8RV29). The male mate choice trials that we recorded were set up identically to the trials that we observed in person, with the exception that each recording could only fit six vials in the frame of the camera (compared with 10 vials that were observed simultaneously by each in‐person observer). We found no significant difference in male courtship preferences (see below) between recorded or in‐person observations for either experiment (Wilcoxon tests; all *p* > .05). As such, we pooled all observations, irrespective of replicate or observation method, for analysis.

### 2.6 Data processing and analysis

We used the courtship data collected above to calculate a “preference index” for each male that measures the strength of the courtship preference for the large female. The preference index (PI) for each male was calculated as:

$$\text{PI}=\frac{\left(\#\;\text{minutes with large female courtship}\right)‐\left(\#\;\text{minutes with small female courtship}\right)}{\text{total}\;\#\;\text{minutes with courtship}}$$

The PI ranges from −1 to +1, with a PI greater than 0 indicating a male preference for the large female (i.e., the male spent more time courting the large female), a PI less than 0 indicating a preference for the small female (i.e., the male spent more time courting the small female), and a PI = 0 indicating no preference (i.e., the male courted the small and large female equally). PI also measures the strength of male mate choice for a specific female: a PI with a higher absolute value indicates a stronger preference for the large female (if PI is positive) or small female (if PI is negative) compared with a PI with a lower absolute value. We performed two analyses using PI for each experiment. We first tested whether or not PIs within each treatment differed significantly from 0 using one‐sample Wilcoxon signed‐rank tests followed by sequential Bonferroni corrections for multiple comparisons (Holm, 1979). We then tested for a significant difference in PI between treatments using a Wilcoxon test (for Experiment 1) or a Kruskal–Wallis test (for Experiments 2 through 4).

Because PI calculates the excess courtship directed towards the large female, we feel it is the most appropriate metric of male mate choice. However, it is possible that males with different sexual histories modified the absolute amount of courtship they performed, which cannot be captured with a preference index. As such, we also calculated the courtship effort directed toward the large female for each male as:

$$\text{Large female courtship effort}=\frac{\#\;\text{minutes with large female courtship}}{\text{total}\;\#\;\text{courtship minutes possible}}$$

For males that did not mate during the male mate choice trials, the total number of courtship minutes possible was 30, and for males that did mate it was the number of minutes until mating began. We tested for significant differences in the large female courtship effort between treatments for each experiment using Wilcoxon/Kruskal–Wallis tests.

We focus on courtship metrics (PI and large female courtship effort) as our main measures of male mate choice because they can quantify the effort that males invest in each female. Metrics that involve mating (such as mating success and/or mating latency with large females) might be more strongly influenced by female mate choice. This is particularly relevant for our study, as *D*. *melanogaster* females have been shown to discriminate between males with different mating histories (Markow et al., 1978; Saleem et al., 2014). Nevertheless, mating is at least partly determined by male mate choice, so we included two mating parameters to complement our courtship analyses: female mated and courtship threshold to mate. First, we tested whether males within each treatment had a mating bias toward large or small females using binomial tests followed by sequential Bonferroni corrections for multiple comparisons (Holm, 1979). Next, we tested for an association between sexual experience treatment and whether the male mated with the large female versus the small female using Pearson's chi‐square tests. Finally, we compared the courtship threshold to mate (measured as the number of minutes a male spent courting a female before mating with her) for both large and small females between treatments using a *t*‐test (for Experiment 1) or one‐way ANOVA (for Experiments 2 through 4). All analyses were performed using JMP 14.

## 3 RESULTS

### 3.1 Experiment 1: Mating with random‐sized females 2 days before male mate choice trials

**FIGURE 1:**
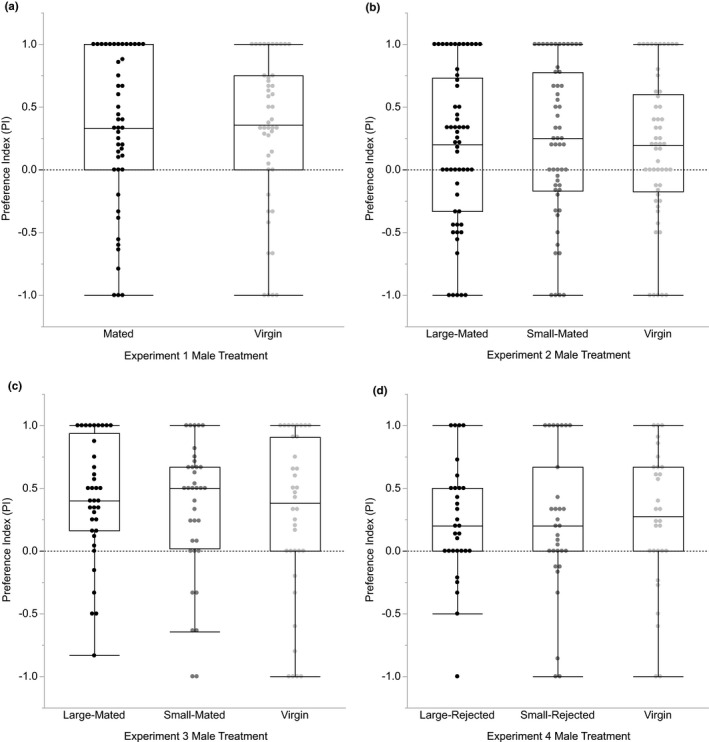
Preferences indices (PIs) for males with different sexual histories. (a) PIs for Experiment 1, in which virgin males were compared with males that had mated with random‐sized females 2 days prior. Both male treatments showed a significantly positive PI (both *p* < .002; *N* = 46–47), indicating that the majority of males spent more time courting the large female compared with the small female, and there was no difference in PI between treatments (*p* = .91). (b) PIs for Experiment 2, in which virgin males were compared with males that had mated with small females or large females 2 days prior. All male treatments showed a significantly positive PI (all *p* < .05; *N* = 54–57) and there were no differences in PIs between treatments (*p* = .88). (c) PIs for Experiment 3, in which virgin males were compared with males that had mated with a small female or a large female immediately before the trials. All male treatments showed a significantly positive PI (all *p* < .02; *N* = 36–37) and there were no differences in PIs between treatments (*p* = .72). (d) PIs for Experiment 4, in which inexperienced virgin males were compared with males that had been rejected by a small female or a large female immediately before the trials. All male treatments showed a significantly positive PI (all *p* < .02; *N* = 30–31) and there were no differences in PIs between treatments (*p* = .91). The dashed horizontal line in each panel indicates the value at which males courted the large and small female equally (i.e., PI = 0; no preference for either the large or small female)

**FIGURE 2:**
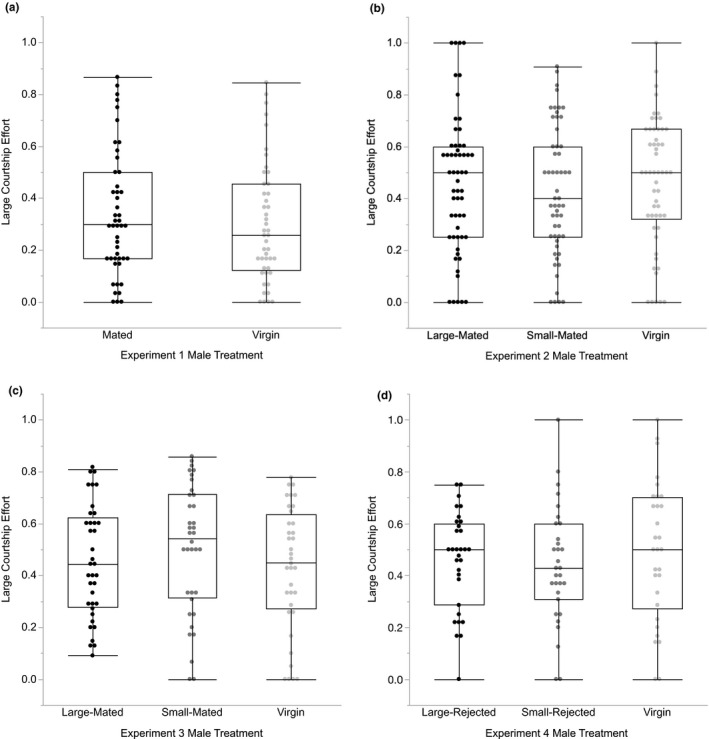
Large female courtship efforts for males with different sexual histories. (a) Large female courtship efforts for Experiment 1, in which virgin males were compared with males that had mated with random‐sized females 2 days prior. There was no difference in courtship effort with large females between treatments (*p* = .46). (b) Large female courtship efforts for Experiment 2, in which virgin males were compared with males that had mated with small females or large females 2 days prior. There were no differences in courtship efforts with large females between treatments (*p* = .72). (c) Large female courtship efforts for Experiment 3, in which virgin males were compared with males that had mated with small females or large females immediately before the trials. There were no differences in courtship efforts with large females between treatments (*p* = .39). (d) Large female courtship efforts for Experiment 4, in which inexperienced virgin males were compared with males that had been rejected by small females or large females immediately before the trials. There were no differences in courtship efforts with large females between treatments (*p* = .58)

We found significantly positive median PIs for virgin males and males that had mated 2 days before male mate choice trials (Figure 1a), indicating that the majority of males from both treatments spent significantly more time courting the large female compared with the small female (Wilcoxon signed‐rank tests; mated: *df* = 46, *p* = .0008; virgin: *df* = 45, *p* = .0016; both *p*‐values were significant after sequential Bonferroni correction). We found no significant differences between treatments in PI (Wilcoxon test: *χ* ^2^ = 0.0126, *df* = 1, *p* = .91) or courtship effort directed toward the large female (Wilcoxon test: *χ* ^2^ = 0.5570, *df* = 1, *p* = .46; Figure 2a), indicating that mated males and virgin males do not differ in the strength of their courtship preference for large females. We similarly found no association between treatment and the type of female mated (Table 1) or any differences between treatments in the courtship thresholds to mate with large females or small females (Table 2), so mated and virgin males do not differ in their mating success with large females.

**TABLE 1:** The propensity to mate with large females compared with small females

Experiment	Treatment	*N*	Proportion of males that mated with large or small females		
			Large female	Small female	Chi‐square test
1	Virgin	46	0.714 (25/35)*	0.286 (10/35)	*χ* ^2^ = 0.115, *p* = .734
	Mated	47	0.750 (27/36)**	0.250 (9/36)	
2	Virgin	54	0.481 (26/54)	0.519 (28/54)	*χ* ^2^ = 4.96, *p* = .084
	Small‐mated	55	0.692 (36/52)**	0.308 (16/52)	
	Large‐mated	57	0.554 (31/56)	0.446 (25/56)	
3	Virgin	36	0.657 (23/35)	0.343 (12/35)	*χ* ^2^ = 1.12, *p* = .57
	Small‐mated	36	0.743 (26/35)**	0.257 (9/35)	
	Large‐mated	37	0.765 (26/34)**	0.235 (8/34)	
4	Virgin/inexperienced	30	0.633 (19/30)	0.367 (11/30)	*χ* ^2^ = 0.906, *p* = .64
	Small‐rejected	31	0.548 (17/31)	0.452 (14/31)	
	Large‐rejected	31	0.516 (16/31)	0.484 (15/31)	

Note

Shown are the mating data for Experiments 1 through 4, including: the total number of males observed during the male mate choice trials for each treatment (*N*), the proportion (and raw counts) of males that mated with the large female, and the proportion (and raw counts) of males that mated with the small female. Male treatments that showed a significant bias toward mating with one type of female over the other are marked with asterisks (binomial tests: *p < .05, **p < .01, all *p*‐values remained significant after sequential Bonferroni correction). Also included for each experiment are the results of a chi‐square test examining whether there is an association between male sexual experience treatment and mating with a large female.

**TABLE 2:** Courtship threshold to mate with large or small females

Experiment	Treatment	Courtship threshold to mate large female			Courtship threshold to mate small female		
		*N*	Mean # min (95% CI)	*t*‐test/ANOVA	*N*	Mean # min (95% CI)	*t*‐test/ANOVA
1	Virgin	25	5.04 (3.67–6.41)	*t* = 0.08, *df* = 50, *p *= .94	10	4.60 (2.14–7.06)	*t* = 0.68, *df* = 17, *p *= .50
	Mated	27	4.96 (3.64–6.28)		9	3.44 (0.85–6.03)	
2	Virgin	26	3.88 (3.04–4.73)	*F*_2,90_ = 0.0235, *p *= .98	28	3.54 (2.66–4.41)	*F*_2,66_ = 0.4698, *p *= .63
	Small‐mated	36	3.78 (3.06–4.50)		16	4.00 (2.84–5.16)	
	Large‐mated	31	3.87 (3.10–4.64)		25	3.28 (2.35–4.21)	
3	Virgin	23	4.87 (3.38–6.36)	*F*_2,72_ = 0.0651, *p *= .94	12	3.17 (1.18–5.16)	*F*_2,26_ = 0.5958, *p* = .56
	Small‐mated	26	4.96 (3.56–6.36)		9	4.78 (2.48–7.08)	
	Large‐mated	26	4.62 (3.22–6.01)		8	3.75 (1.31–6.19)	
4	Virgin/inexperienced	19	5.05 (3.70–6.41)	*F*_2,49_ = 0.3641, *p *= .70	11	3.45 (2.42–4.49)	*F*_2,37_ = 0.5515, *p* = .58
	Small‐rejected	17	4.24 (2.80–5.67)		14	3.64 (2.73–4.56)	
	Large‐rejected	16	4.50 (3.02–5.98)		15	3.00 (2.11–3.89)	

Note

Shown are the data for Experiments 1 through 4. Courtship threshold to mate with a large (or small) female was measured as the number of minutes a male spent courting a large (or a small) female during the male mate choice trials before he mated with that large (or small) female. Shown for each experiment are the results of a *t*‐test or ANOVA testing for a difference between treatments in the courtship thresholds to mate with large or small females.

### 3.2 Experiment 2: Mating with small or large females 2 days before male mate choice trials

Consistent with our results from Experiment 1, we found significantly positive median PIs for all three male treatments, indicating that most virgin (Wilcoxon signed‐rank test; *df* = 53, *p* = .022), small‐mated (Wilcoxon signed‐rank test; *df* = 54, *p* = .0069), and large‐mated (Wilcoxon signed‐rank test; *df* = 56, *p* = .0453) males courted the large female more than the small female (all *p*‐values significant after sequential Bonferroni correction; Figure 1b). When we compared between treatments, we found no difference in PI (Kruskal–Wallis test: *χ* ^2^ = 0.2478, *df* = 2, *p* = .88) or courtship effort with the large female (Kruskal–Wallis test: *χ* ^2^ = 0.6658, *df* = 2, *p* = .72; Figure 2b), so mating experience 2 days prior did not affect the strength of male courtship preferences for large females. As with Experiment 1, mating with the large female was not associated with sexual experience (Table 1), and the courtship thresholds to mate with the large female or the small female did not differ between treatments (Table 2).

### 3.3 Experiment 3: Mating with small or large females immediately before male mate choice trials

When we tested for an effect of mating immediately before male mate choice trials, we again found that all male treatments had significantly positive median PIs (Wilcoxon signed‐rank tests; virgin: *df* = 35, *p* = .0192; small‐mated: *df* = 35, *p* = .0008; large‐mated: *df* = 36, *p* < .0001; all *p*‐values significant after sequential Bonferroni correction) and that there were no differences between treatments in PI (Kruskal–Wallis test: *χ* ^2^ = 0.6533, *df* = 2, *p* = .72; Figure 1c) or in the courtship effort directed toward the large female (Kruskal–Wallis test: *χ* ^2^ = 1.8824, *df* = 2, *p* = .39; Figure 2c). Finally, there was no relationship between sexual experience treatment and the type of female mated (Table 1), nor were there any differences between treatments in the courtship thresholds to mate with the large female or the small female (Table 2).

### 3.4 Experiment 4: Rejection by small or large females immediately before male mate choice trials

Finally, we tested whether being rejected by a small or large female affected the strength of male mate choice and found no differences in PI (Kruskal–Wallis test: *χ* ^2^ = 0.1976, *df* = 2, *p* = .91; Figure 1d) or courtship effort with the large female (Kruskal–Wallis test: *χ* ^2^ = 1.0922, *df* = 2, *p* = .58; Figure 2d) between our three male treatments. As with our previous experiments, we found significantly positive median PIs for our large‐rejected (Wilcoxon signed‐rank test; *df* = 30, *p* = .0041), small‐rejected (Wilcoxon signed‐rank test; *df* = 30, *p* = .0179), and inexperienced virgin male treatments (Wilcoxon signed‐rank test; *df* = 29, *p* = .0148), indicating that the majority of males from all treatments courted the large female more than the small female (all *p*‐values significant after sequential Bonferroni correction). We again found that sexual experience was not associated with whether the male mated with the large or small female (Table 1), and the courtship thresholds to mate with the large female or the small female did not differ between treatments (Table 2).

## 4 DISCUSSION

In this study, we investigated the potential impact that previous sexual experiences may have on the strength of *D*. *melanogaster* male mate choice for large‐bodied females. We found that sexually inexperienced males had a robust courtship preference for large females in all four experiments, consistent with past work using a different population of *D*. *melanogaster* (Baxter et al., 2015). Despite this courtship preference, virgin males only mated with significantly more large females than small females in Experiment 1 (Table 1). The inconsistency of these mating data might reflect variation in female mate choice between experiments, which is beyond the scope of this study. Nevertheless, virgin males show similar preferences for larger females in other insect species, including lesser wax moths (Goubault & Burlaud, 2018), seaweed flies (Pitafi et al., 1990), stink bugs (Capone, 1995), and bedbugs (Kaufmann & Otti, 2019). In *D*. *melanogaster*, sexually inexperienced males also exhibit courtship or mating preferences based on female fecundity (Edward & Chapman, 2012), infection status (Khan & Prasad, 2013), mating history (Siegel & Hall, 1979), and species (Shahandeh et al., 2020). Although experimentally elevating male costs (e.g., through sperm/ejaculate depletion, Byrne & Rice, 2006; Nandy et al., 2012) might increase the strength of male choosiness, this is clearly not required for male mate choice to occur. Our findings therefore add to a growing body of literature demonstrating that sexually inexperienced males can be quite choosy with respect to specific female traits.

After documenting a robust courtship preference for large females in virgin males, we found no difference in the strength of this preference when compared with males from any of our mating experience treatments. This result was consistent regardless of the body size of the female first mated, when the sexual experience occurred, or the metric used to measure male mate choice. This finding contrasts with the plasticity seen in male courtship preferences based on female species and mating status. When compared with virgin inexperienced males, males that had previously mated with a single conspecific female courted heterospecific females less, males that had mated with a single virgin female courted mated females less, and males that had mated with a single simulated‐mated female (i.e., a virgin female coated in the pheromones of mated females) courted mated females more (Dukas & Dukas, 2012). A single mating experience is clearly sufficient to modify male courtship behavior toward mated and heterospecific females, but this does not seem to be the case for male mate choice based on female body size.

Mating is not the only sexual experience that could potentially modify male mate choice. A single rejection experience has been shown to alter *D*. *melanogaster* male courtship preferences based on female age (Balaban‐Feld & Valone, 2018), mating status (Dukas & Dukas, 2012), and species (Dukas, 2004; Dukas & Dukas, 2012). Despite this, past rejection by a small or large female did not affect any metric of male mate choice for large females in our study. It is worth noting that we used nonvirgin small and large females as our “rejecting females,” which likely exposed males to multiple female cues during the experience phase (i.e., the mating status and body size of the rejecting female). Nevertheless, this is a realistic rejection experience, as non‐receptive *D*. *melanogaster* females are usually those that have previously mated (Wolfner, 1997). Additionally, our results are comparable to work from Balaban‐Feld and Valone (2018), who used large or small decapitated virgin females as rejecting females, but still found no effect of this rejection on male courtship persistence with subsequent large or small females.

The finding that a single mating or rejection experience did not affect the strength of male mate choice for large females was surprising given the plasticity based on sexual experience that has been reported for other male preferences in this species (Balaban‐Feld & Valone, 2018; Dukas, 2004; Dukas & Dukas, 2012; Ejima et al., 2005). One possibility is that the preference seen in virgin males is too strong to be altered by sexual experience. The strength of male mate choice correlates positively with the difference in female fecundity in *D*. *melanogaster* (Edward & Chapman, 2012; Nandy et al., 2012), and the large females used here are approximately twice as fecund as the small females (Byrne & Rice, 2006). However, work by Edward and Chapman (2012) similarly found that male mating status did not affect the strength of male mate choice for higher fecundity females when using females with much smaller fecundity differences than those in our study. Although Edward and Chapman (2012) did not explicitly consider female body size, male mate choice for traits associated with female fecundity may be relatively unaffected by sexual experience, potentially underscoring the adaptive nature of these preferences.

It is possible that additional forms of experience, such as multiple mating and/or rejection episodes, might have affected the strength of male mate choice for large females compared with inexperienced males. However, the goal of our study was not to test whether any form of experience could change this preference, but instead whether common forms of sexual experience (i.e., a single mating or rejection experience) that produce plasticity in other male preferences would similarly affect this preference. An important consideration of our study, however, is that males only encountered a non‐competitive environment for both the sexual experience phase and the male mate choice trials. Although non‐competitive conditions were also used in studies that found an effect of experience on other male preferences (Balaban‐Feld & Valone, 2018; Dukas, 2004; Dukas & Dukas, 2012; Ejima et al., 2005), courtship plasticity might be more strongly favored in a male–male competitive environment if males are able to use their experience to gain information about their expected future success with large females relative to other males (as predicted by Fawcett & Bleay, 2009).

Finally, our methods for creating small females (limiting food resources during the larval stage) might affect additional components of female condition. If these effects are substantial enough that small females respond abnormally to male courtship, males might direct more courtship toward the large female regardless of their mating history. We think this is unlikely because of the data from the mating experience phase of Experiment 3. When males were paired with a single large virgin female or a single small virgin female, there was no difference in the number of large versus small females mated, or in the time it took males to start mating with the large or small females (see Materials and Methods for Experiment 3). The fact that males were readily able to court and mate with small females in a no‐choice environment indicates that small females were acceptable courtship targets.

Our study has shown that male mate choice for large‐bodied females in *D*. *melanogaster* does not differ between virgin males and males that had mated or been rejected previously. Our findings are consistent with past work documenting male mate choice for large females in this species (Byrne & Rice, 2006; Dukas & Baxter, 2014; Long et al., 2009), but differ from studies that demonstrated experience‐based plasticity for other male preferences (Balaban‐Feld & Valone, 2018; Dukas, 2004; Dukas & Dukas, 2012; Ejima et al., 2005). The mechanisms underlying these differences in plasticity remain unclear, signifying that we still have much to learn about how male preferences develop, the degree to which they are adaptive, and their overall contribution to sexual selection within a population.

## CONFLICT OF INTEREST

The authors do not have any conflicts of interest to declare.

## AUTHOR CONTRIBUTIONS

**Chelsea S. Sinclair:** Formal analysis (equal); investigation (equal); visualization (equal); writing‐original draft (equal); writing‐review & editing (equal). **Suriya F. Lisa:** Investigation (supporting); writing‐review & editing (equal). **Alison Pischedda:** Conceptualization (lead); formal analysis (equal); funding acquisition (lead); investigation (equal); methodology (lead); supervision (lead); visualization (equal); writing‐original draft (equal); writing‐review & editing (equal).

## Data Availability

Data referenced in this study are available through Dryad: https://doi.org/10.5061/dryad.1g1jwstxq.
